# circTP63 promotes prostate cancer progression via miR-421/VAMP associated protein A axis

**DOI:** 10.7150/jca.99561

**Published:** 2024-08-19

**Authors:** Jianfeng Xu, Siwei Xu, Weihui Liu, Jiabi Chen, Longbo Cai, Wei Zhuang

**Affiliations:** 1 Department of Urology, Jinjiang Municipal Hospital. No. 16, Luoshan Section, Jinguang Road, Luoshan Street, Jinjiang City, Quanzhou, Fujian, China.; 2 Department of Urology, The Second Affiliated Hospital of Fujian Medical University, Quanzhou 362000, Fujian, China.

**Keywords:** prostate cancer, circTP63, miR-421, VAPA

## Abstract

**Background:** Circular RNAs (circRNA) have a vital role in the progression of cancers. For instance, circTP63 is upregulated in prostate cancer (PCa) tissues compared with adjacent normal tissues. However, the role of circTP63 in prostate cancer is still unclear.

**Methods:** qRT-PCR assays were applied to detected the expression of circTP63 and miR-421 in PCa samples. Functionally, CCK-8, apoptosis assay, and transwell migration and invasion assays were used to explore the role of circTP63 in PCa progression. Mechanistically, the interaction between circTP63 and miR-421 were verified using qRT-PCR and dual-luciferase report assay. Western blot, qRT-PCR, and dual-luciferase report assay were applied to detect the interaction between miR-421 and VAMP associated protein A (VAPA). And xenograft animal model was used to detect the role of circTP63 *in vivo*.

**Results:** circTP63 was upregulated and miR-421 was downregulated in PCa tissues. Functional assays revealed that circTP63 promoted the proliferation and metastasis of PCa cells *in vitro*. In addition, the inhibition effect of circTP63 knockdown could be rescued by miR-421 inhibition or VAPA overexpression. Mechanistically, circTP63-mediated PCa progression through directly binding to miR-421, and subsequently releasing the VAPA.* In vivo,* silencing of circTP63 significantly impaired PCa progression.

**Conclusion:** In summary, our study identified circTP63 as an oncogenic circRNA, which could be a promising diagnostic and therapeutic target for PCa treatment.

## Introduction

Prostate cancer is the most common tumor in men in the USA 1. Androgen deprivation therapy (ADT) is currently the standard therapeutic strategy for PCa treatment, as androgen receptor (AR) plays an indispensable role in PCa progression 2,3. However, most patients with prostate cancer become resistant after 1 to 2 years of treatment 4. Therefore, exploring novel therapeutic targets for prostate cancer is of high urgency.

Circular RNA is a recently discovered class of noncoding RNA that is produced by the back-splicing process 5. Although it was originally considered as an error in normal splicing events, it was later considered to have a possible function 6. In recent years, with the development of high throughout sequencing and specific algorithms, thousands of circRNAs were identified 7. circRNAs have been reported to be involved in various biological processes through serving as miRNAs sponges, interacting with RNA binding proteins, and their translation 8,9.

Several TP63-derived circRNAs have been shown to be involved in the progression of cancer. Hsa_circ-0068515, derived from TP63, was found to promote lung squamous cell carcinoma progression via sponging miR-873-3p and preventing FOXM1 degradation 10. Another group revealed that circTP63 facilitated gastric cancer progression by sponging miR-217, further upregulated oncogene EZH2 expression 11. Wang et al. reported that circTP63 promoted the progression of liver cancer via interacting with miR-155-5p and eventually elevating the expression of ZBTB18 12. Meanwhile, circTP63 was found to promote breast cancer progression through miR-873-3p/FOXM1 axis 13. However, the role of circTP63 in prostate cancer is still largely unexplored.

It has been recognizing that miR-421 as a promising target for cancer diagnosis and therapy. Studies have shown that miR-421 can directly target PTEN, leading to the activation of PI3K/AKT/mTOR pathway and promoting lung cancer progression 14. Surprisingly, miR-421 could be secreted by cancer-associated fibroblast to promote pancreatic cancer proliferation by regulating the SIRT3/H3K9Ac/HIF-1α axis 15. Furthermore, miR-421 have been shown to suppress ATM expression, contributing to prostate cancer progression 16. However, despite these findings, the full spectrum of miR-421's role in prostate cancer remains to be elucidated.

VAPA is an endoplasmic reticulum (ER) integral protein that serves to recruit lipid-binding proteins to the ER membrane 17. It could bind to several lipid transfer proteins, which contains the two phenylalanines in an acidic tract (FFAT) motif, such as CERT, ORP3, and ORP1L 18. VAPA is reported to function in maintaining ER membranes, vesicle trafficking, and regulating the microtubule network 19-21. Notably, VAPA is highly enriched in small RNA-containing vesicles. Functionally, VAPA knockout (VAPA-KO) cells inhibit colon cancer progression by reducing the transfer of specific RNAs 22. Conversely, VAPA fusion with Rab31 significantly enhances anti-apoptotic pathway via upregulating BCL-2 expression, promoting lung cancer progression 23. However, the existing knowledge on its role in prostate cancer is still limited.

The goal of this study was to investigate the role of circTP63/miR-421/VAPA axis in prostate cancer. As circTP63 was previously found to be upregulated in prostate cancer tissues compared with normal tissues. Thus, we hypothesized that circFP63 may promote prostate cancer progression. Functional assays revealed that silencing of circTP63 inhibited PCa proliferation and metastasis. Next, we aimed to explore the detailed mechanism of circTP63 promoting PCa progression. The results showed that circTP63 could competitively bind to miR-421 and finally increase the expression of VAPA. Overall, we suggest that circTP63 could be a potential diagnostic and therapeutic target for PCa.

## Material and methods

### Patients characteristics

A total of 40 paired prostate cancer and normal tissues were accessed from the PCa patients at The Second Affiliated Hospital of Fujian Medical University, and the informed consents were obtained. All the patients were diagnosed with PCa, which confirmed by histopathological examination. We excluded patients who underwent androgen deprivation treatment, radiotherapy or chemotherapy before surgery and those with severe underlying diseases. This study was approved by the Ethics Committee of The Second Affiliated Hospital of Fujian Medical University (2022-273). The detailed information of patients was listed in Supplementary file, “Clinical information of patients”.

### Cell culture

The human prostate cancer cell lines LNCap, C4-2, DU145, and PC-3 were purchased from the American Type Culture Collection (ATCC). The DU145, LNCap, and C4-2 cell lines were cultured in RPMI-1640 with 10% fetal bovine serum (FBS). PC-3 was cultured in MEM medium with 10% FBS.

### Cell transfection

miR-421 mimics, inhibitors, and siRNAs for circTP63 were synthesized by GenePharma (Shanghai, China). Lipofectamine 3000 (Invitrogen, USA) was used to transfect miR-421 mimics, inhibitors, siRNAs, and plasmids to DU145 and PC-3 cells.

### qRT-PCR

Total cells and tissues RNA were extracted by the Trizol reagent (Invitrogen, USA). Then, the RNAs were reverse-transcribed to cDNA using the PrimeScript RT Reagent Kit (Takara, Japan). qRT-PCR was performed using a SYBR Green PCR Kit (Takara, Japan). U6 and GAPDH were used as endogenous control for miRNA and mRNA respectively. The primer sequences were presented in Supplementary Table S1.

### Western blot

Briefly, the PCa cells were lysed in RIPA lysis buffer. Subsequently, the lysates were separated using SDS-PAGE and transferred onto PVDF membranes (Millipore, USA). Then, the membranes were incubated with the primary antibodies: anti-VAPA (Abcam, ab181067, 1:1000), and anti-GAPDH (Abcam, ab8245, 1:1000) at 4°C overnight. Then, the membrane was incubated with secondary antibodies for 1 hour. The ImageJ software was used to analyze the results.

### Transwell assays

The transwell assays were conducted by using transwell chamber (Corning, NY, USA) according to the manufacturers' instructions. Briefly, 2x10^4^ transfected PCa cells were place in the upper chamber with or without Matrigel using FBS-free medium, while those in the lower chamber placed with medium with 10% FBS. After 24h, the chamber was fixed and stained with 0.3% crystal violet. Finally, the chamber was imaged and the migrated cell numbers were counted.

### Flow cytometry

Cell apoptosis was detected using Annexin V-PI Apoptosis Detection Kits (BD Biosciences, USA). Briefly, after transfected with circTP63 siRNAs or overexpression plasmids, the PCa cells were digested and stained with Annexin V-PI for 15 min. Then, the apoptosis rate was detected using flow cytometry.

### RNA immunoprecipitation

The RIP assay was performed using the Protein Immunoprecipitation Kit (Millipore, MA, USA) according to the manufacturers' instruction. The detailed protocol has been reported previously 24. Briefly, the magnetic beads were conjugated with AGO2 or IgG antibodies at room temperature for 30min and then incubated with cell lysates overnight. After 5 times of washing, the protein was digested with proteinase K, and the precipitated RNA was detected using qRT-PCR.

### Dual-luciferase report assay

About 1x10^4^ PC-3 cells were plated into the 24-well plates. circTP63 or VAPA wild-type or mutant plasmids and miR-421 mimics or control were transfected into PC-3 cells. After 48 h, cells were harvested and the luciferase activity was measured using a dual-luciferase reporter assay system (Promega, USA). Renilla luciferase was used as internal control.

### Tumor xenograft models in nude mice

The BALB/c nude mice obtained from the Laboratory Animal Center of Fujian Medical University were housed under specific pathogen-free conditions at the Fujian Medical University animal care facility. To detect the effect of circTP63 on the growth of PCa cells *in vivo*, circTP63 stably knockdown or scramble PC-3 cells were constructed. About 1 x 10^7^ PC-3 cells were injected subcutaneously into the nude mice. After 6 weeks, the mice were anaesthetized via cervical dislocation. The tumors in the mice were collected, and their tumor volume and weight were measured. The animal experiments were approved by the Ethics Committee of The Second Affiliated Hospital of Fujian Medical University (2021-437).

### Statistical analysis

All statistical analyses were performed with Prism GraphPad version 8.0 (GraphPad, USA). Data were presented as mean ± SEM. Student's t-test was used to determine the statistical differences between groups. Pearson's correlation coefficient was used for correlation analysis. *P* < 0.05 was considered as indicative of statistical significance.

## Results

### circTP63 is upregulated in prostate cancer tissues and cells

**Figure 1:**
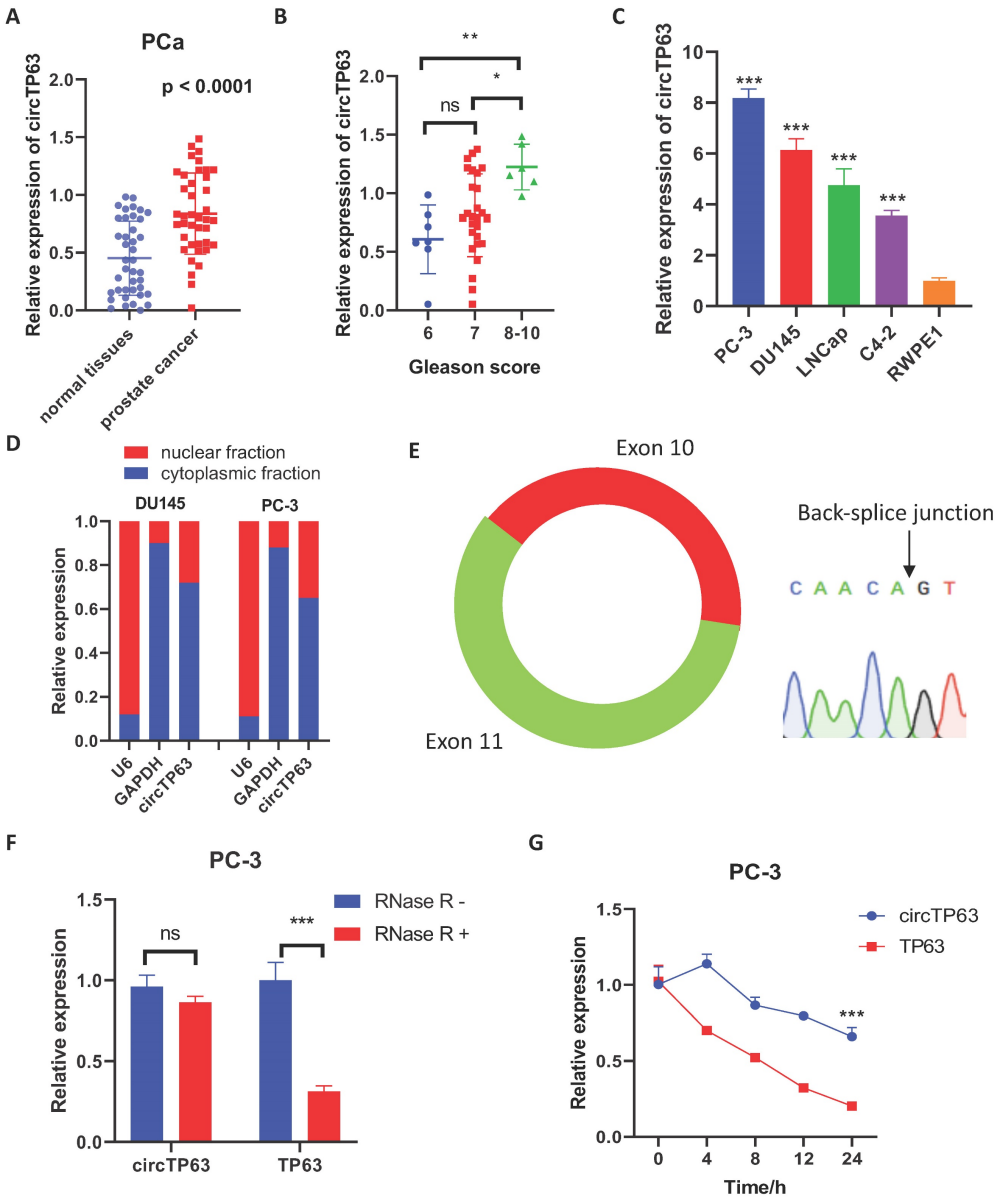
circTP63 is upregulated in prostate cancer. **A,** The relative expression of circTP63 in 40 paired prostate cancer and adjacent normal tissues. B, The relative expression of circTP63 in Gleason score 6, 7, and 8-10 patients. **C,** The relative expression of circTP63 in RWPE-1 and 4 prostate cancer cell lines. **D,** Cellular distribution analysis of circTP63 in PCa cells. **E,** Sanger sequencing showing the sequence of the circTP63 junction site. **F,** The relative expression of circTP63 and TP63 with or without RNase R treatment.** G,** The relative expression of circTP63 and TP63 after Actinomycin D treatment.

Firstly, we evaluated the expression of circTP63 in PCa tissues to identify the expression pattern of circTP63 in prostate cancer. The result demonstrated that circTP63 was upregulated in 40 paired prostate cancer tissues compared with normal tissues (Figure 1A). Also, circTP63 was upregulated in PCa patients with Gleason score more than 9 compared to PCa patients with Gleason score 6 - 8 (Figure 1B) (Table 1). Next, we measured the expression of circTP63 in these cancer cell lines. The results confirmed that circTP63 was upregulated in prostate cancer cell lines (LNCaP, PC-3, C4-2, and DU145) compared with normal prostate epithelial cell RWPE-1 (Figure 1C). Nuclear and cytosol fractionation assay showed that circTP63 was predominantly localized in the cytosol (Figure 1D). Then, we confirmed the qPCR product of circTP63 with divergent primers using Sanger sequencing (Figure 1E). The RNase R digestion experiment demonstrated that circTP63 was more resistant to RNase R treatment compared with TP63 mRNA (Figure 1F). In addition, Actinomycin D assay also confirmed that circTP63 was more stable than TP63 mRNA (Figure 1G). These findings suggested the high-expression and circular structure of circTP63 in prostate cancer.

### Silencing of circTP63 restrained PCa progression *in vitro*

**Figure 2:**
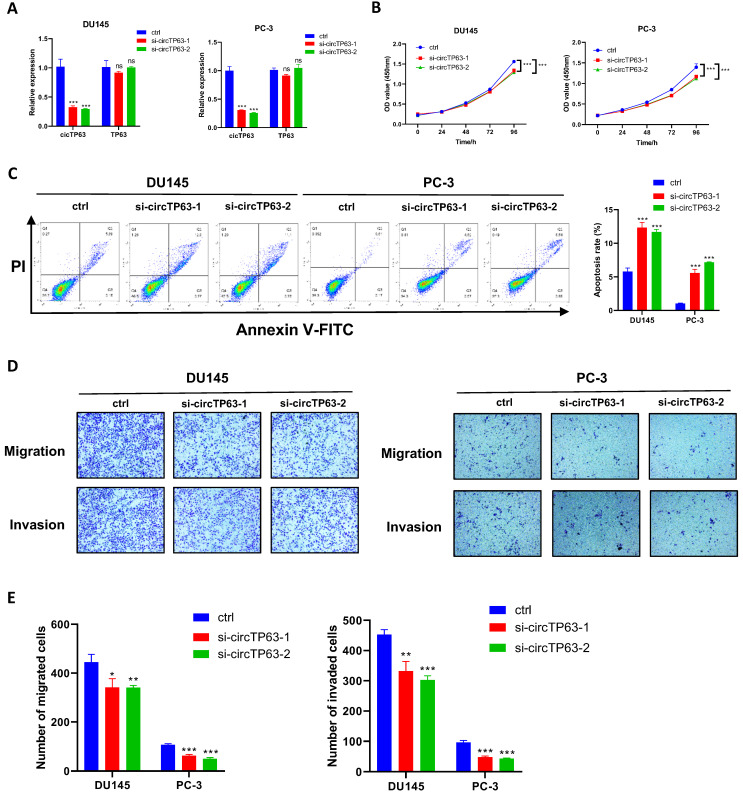
Silencing of circTP63 inhibits PCa progression. **A,** The relative expression of circTP63 and TP63 in DU145 and PC-3 cells transfected with circTP63 siRNAs. **B**, The growth curve of PCa cells evaluated by CCK-8 assay with circTP63 knockdown. **C,** Flow cytometry assays showing the apoptosis rate in PCa cells with circTP63 knockdown. **D, E,** Transwell assay in PCa cells with circTP63 knockdown.

Next, we aimed to determine the functional effect of circTP63 on prostate cancer. Firstly, circTP63 siRNAs, which specifically target the junction site, and overexpression plasmids were designed and synthesized. The circTP63 knockdown and overexpression efficiency was verified using qRT-PCR (Figure 2A and Supplementary Figure 1A). Then, CCK-8 assay revealed that silencing of circTP63 significantly impaired the vitality of DU145 and PC-3 cells (Figure 2B), while overexpression of circTP63 promoted the vitality of C4-2 cells (Supplementary Figure 1B). Moreover, enhanced apoptosis was observed in circTP63 knockdown PCa cells (Figure 2C). In addition, transwell assays demonstrated that silencing of circTP63 restrained the metastasis ability of DU145 and PC-3 cells (Figure 2D and 2E). Moreover, overexpression of circTP63 promoted the metastasis ability of C4-2 cells (Supplementary Figure 1C). We next investigated the role of circTP63 in the normal prostate epithelial cell line, RWPE-1. Since circTP63 expression was downregulated in these cells, we overexpressed circTP63 in RWPE-1. The CCK-8 and colony formation assays revealed that circTP63 overexpression had a minimal effect on RWPE-1 cell proliferation. These findings suggest that circTP63 may play a more significant role in the context of PCa cells, with less impact on normal prostate epithelial cells (Supplementary Figure 1D). In conclusion, these data showed that silencing of circTP63 restrained prostate cancer proliferation and metastasis.

### circTP63 functions as a sponge for miR-421

**Figure 3:**
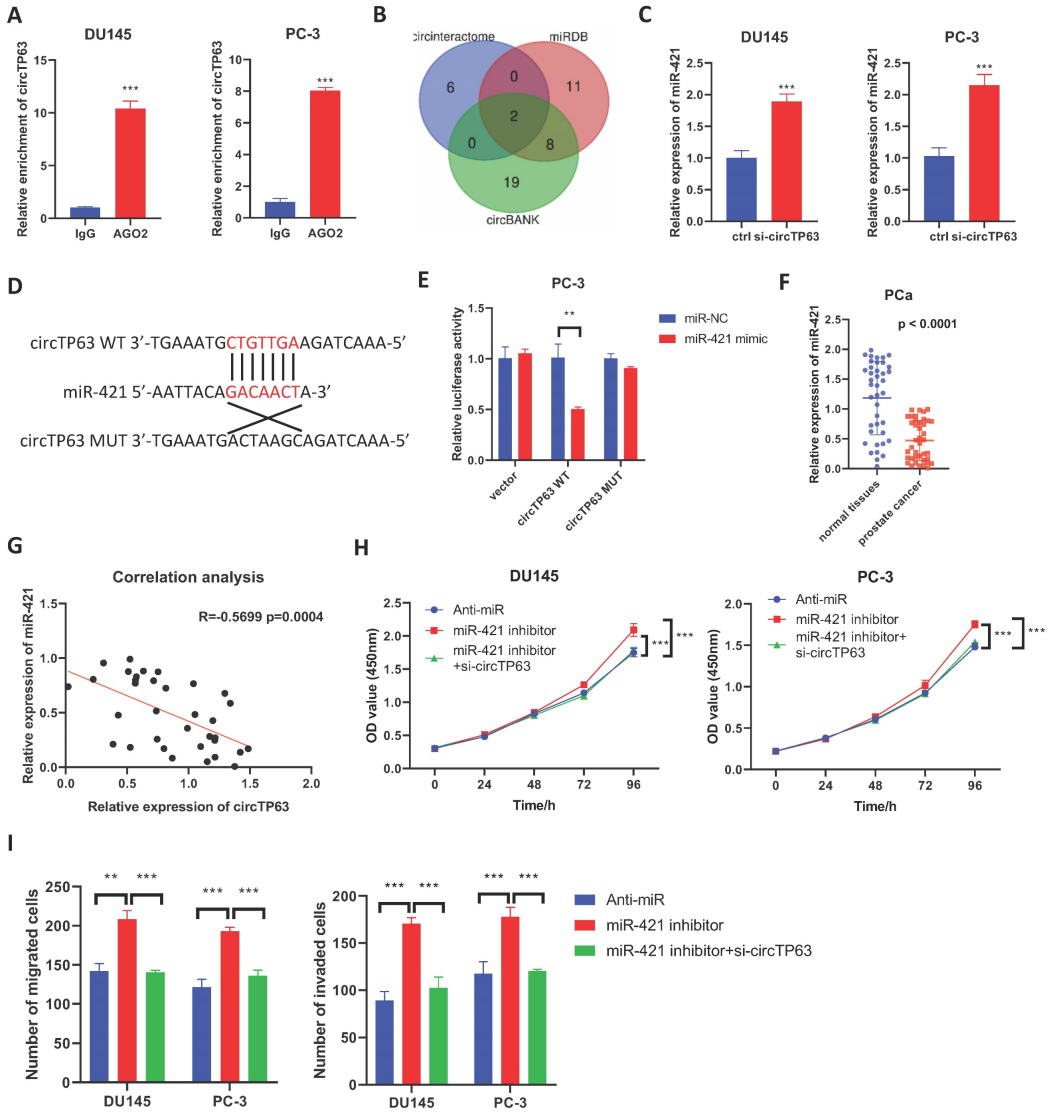
circTP63 directly binds to miR-421. **A,** The enrichment of circTP63 in AGO-RIP assay. **B,** Venn diagram showing the predicted circTP63 interacted miRNAs using miRDB, circBank, and circinteractome. **C,** The relative expression of miR-421 in PCa cells transfected with circTP63 siRNAs. **D,** Predicted binding site of miR-421 in circTP63. **E,** The relative luciferase activity of wild-type or mutant circTP63 in PCa cells with or without miR-421 overexpression. **F,** The relative expression of miR-421 in 40 paired prostate cancer and adjacent normal tissues.** G,** Correlation analysis between miR-421 and circTP63 in 40 prostate cancer tissues. **H,** The growth curve of DU145 and PC-3 with circTP63 knockdown rescued the effect of miR-421 inhibitor.** I,** Transwell assay of DU145 and PC-3 cells with circTP63 knockdown rescued the effect of miR-421 inhibitor.

Since circRNAs often exerts its regulatory role through the ceRNA network, we first performed AGO2-RIP. The results showed that circTP63 could bind to AGO2 both in PC-3 and DU145 cells (Figure 3A), suggesting that circTP63 could serve as a miRNA sponge. Then, bioinformatics analysis was performed using circBank, circinteractome, and miRDB to identify the potential target miRNAs of circTP63 in prostate cancer. The results demonstrated that miR-421 and miR-1206 could be the potential target of circTP63 (Figure 3B). Next, we considered that miR-421 had been extensively explored in prostate cancer as a tumor-suppressive miRNA, while miR-1206 had been rarely studied. Thus, miR-421 was selected as a target miRNA of circTP63. Interestingly, silencing of circTP63 significantly increased the expression of miR-421, indicating that miR-421 might be the downstream target of circTP63 (Figure 3C). Dual-luciferase reporter assay was then performed, which revealed that miR-421 mimics significantly decreased the luciferase activity of wild-type circTP63, while have little effect on mutant circTP63 (Figure 3D and 3E). Next, we evaluated the expression of miR-421 in prostate tissues and the results showed that miR-421 was downregulated in prostate cancer tissues (Figure 3F). Moreover, the expression of circTP63 was negatively correlated with that of miR-421 in prostate cancer tissues (Figure 3G). These data confirmed that miR-421 was the downstream target of circTP63.

Then, CCK-8 and transwell assays were performed to verify the role of miR-421 in prostate cancer. The results showed that the promotion effect of miR-421 in PCa cells' proliferation could be reversed by knockdown of circTP63 in both PCa cells (Figure 3H). Similar results were observed in the transwell assay (Figure 3I). These findings demonstrated that circTP63 promote prostate cancer progression through targeting miR-421.

### VAPA is the downstream target of miR-421

**Figure 4:**
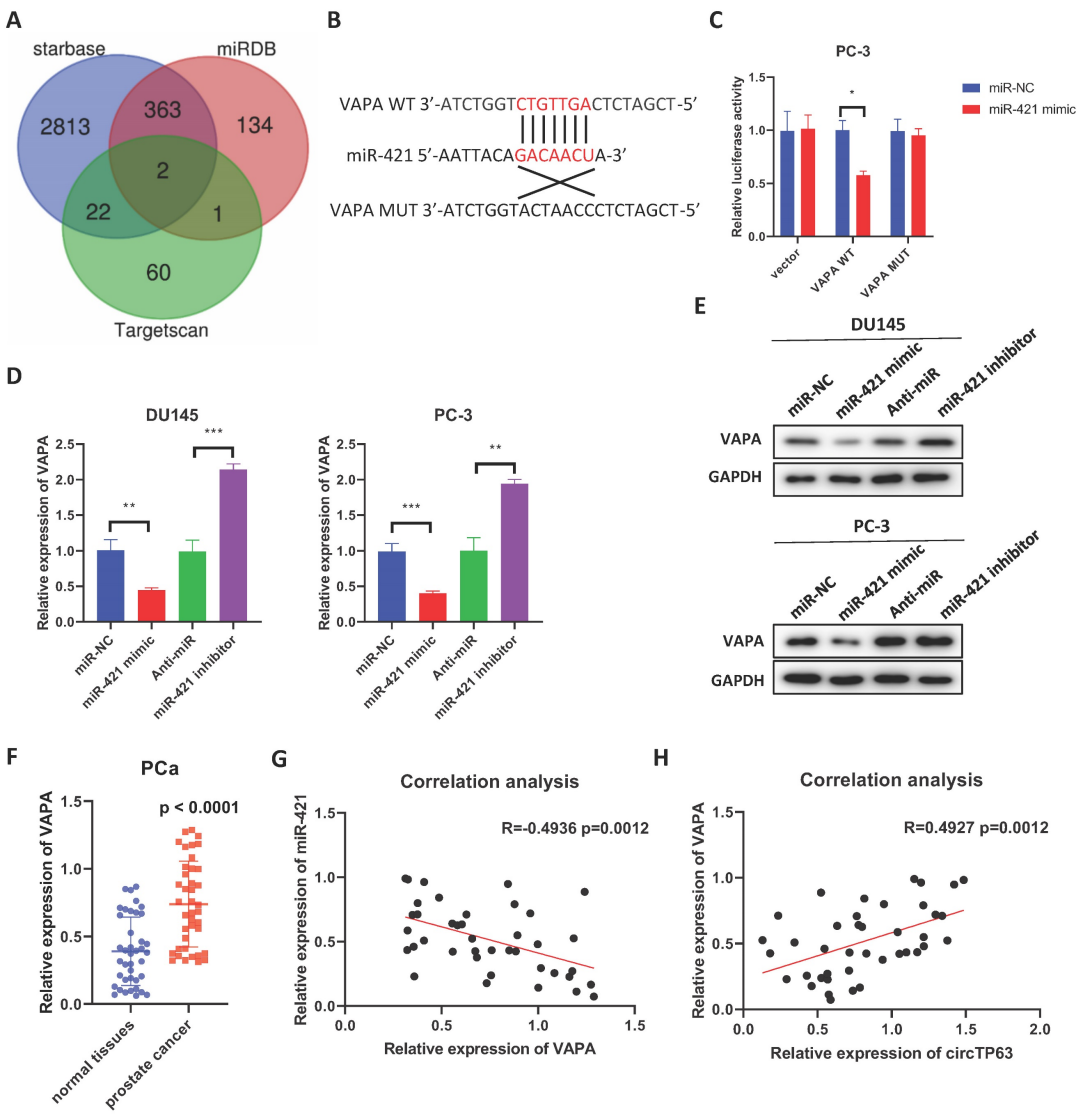
VAPA is the downstream target of miR-421. **A,** Venn diagram showing the predicted miRNA targets using miRDB, starbase, and Targetscan. **B,** Predicted binding site of miR-421 in VAPA. **C,** The relative luciferase activity of wild-type or mutant VAPA in PCa cells with or without miR-421 overexpression. **D, E,** The relative mRNA (**D**) or protein (**E**) expression of VAPA in DU145 and PC3 cells transfected with miR-421 mimics or miR-421 inhibitors. **F,** The relative expression of VAPA in 40 paired prostate cancer and adjacent normal tissues.** G,** Correlation analysis between VAPA and miR-421 in 40 prostate cancer tissues. **H,** Correlation analysis between VAPA and circTP63 in 40 prostate cancer tissues.

In order to figure out the specific target of miR-421, three databases, Starbase, Targetscan, and miRDB were used (Figure 4A). Two potential targets (VAPA and PCNP) were identified, but only the expression of VAPA was upregulated in the TCGA database (PRAD) (Supplementary Figure 2A). Next, dual-luciferase reporter assay was performed, which showed that miR-421 mimics significantly decreased the luciferase activity of wild-type VAPA, while had little effect on mutant VAPA (Figure 4B and 4C). Also, overexpression of miR-421 decreased the expression of VAPA, while silencing of miR-421 increased its expression (Figure 4D and 4E). Moreover, we examined the expression of VAPA in prostate cancer tissues. The results demonstrated that VAPA was upregulated in prostate cancer tissues compared with normal tissues (Figure 4F). Furthermore, the expression of VAPA was negatively correlated with that of miR-421 in prostate cancer tissues (Figure 4G), and it was positively correlated with that of circTP3 in prostate cancer tissues (Figure 4H). We then explored whether inhibition of miR-421 promoted PCa progression through upregulating VAPA expression. CCK-8 assays showed that miR-421 inhibition promoted PCa cells proliferation, while this effect could be rescued by VAPA knockdown (Supplementary Figure 3A). Moreover, the transwell assays had the same results (Supplementary Figure 3B). Overall, these findings showed that VAPA is a downstream target of miR-421.

### circTP63 promotes prostate cancer progression through VAPA

**Figure 5:**
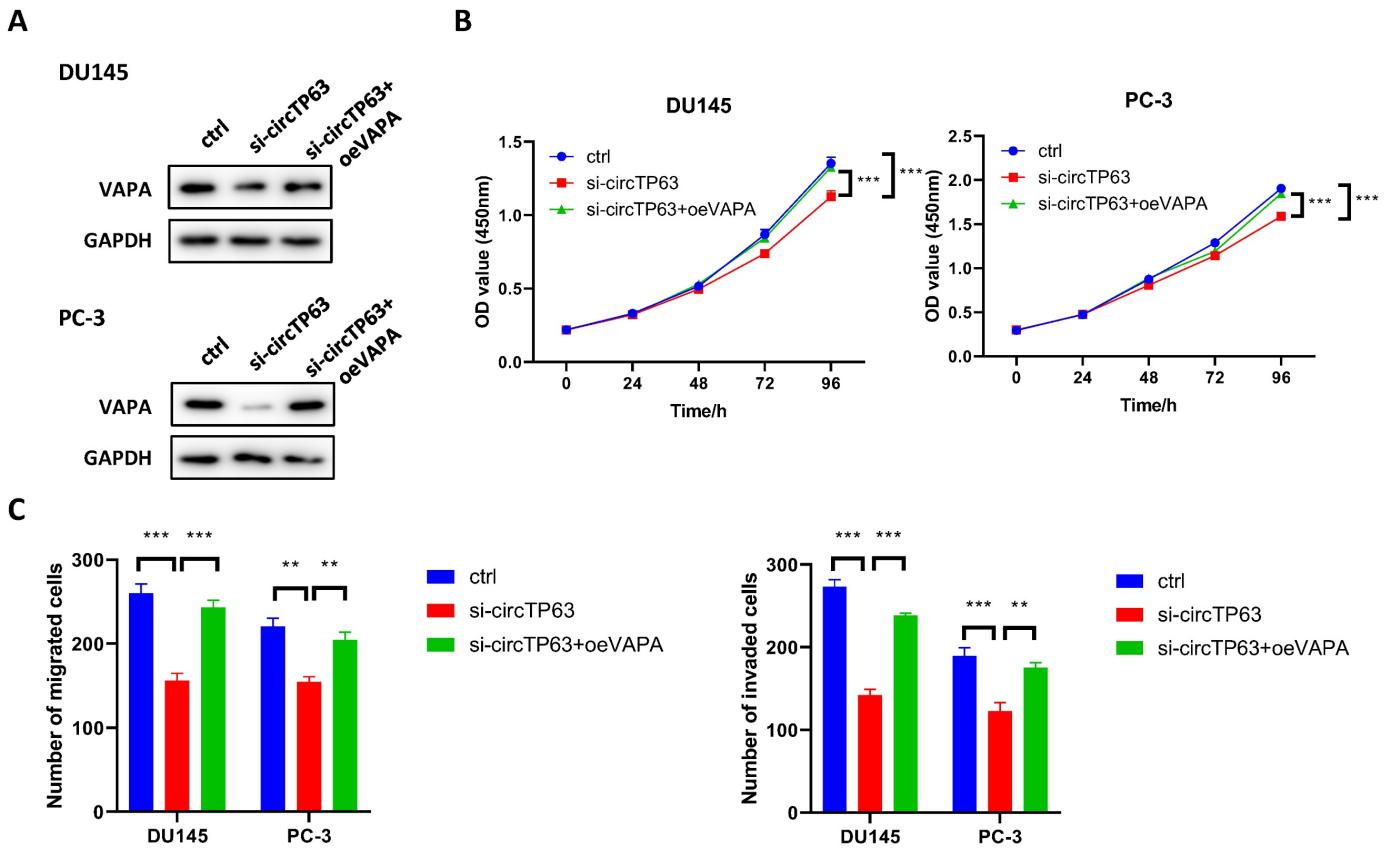
circTP63 promotes PCa progression via VAPA. **A,** The expression of VAPA in circTP63 knockdown PCa cells with VAPA overexpression. **B,** The growth curve of PCa cells with VAPA overexpression that reversed the inhibition effect of circTP63 silencing. **C,** Transwell assay of DU145 and PC-3 with VAPA overexpression that reversed the inhibition effect of circTP63 silencing.

We next explored whether circTP63 promoted PCa progression through regulating VAPA expression. Western blot assay demonstrated that silencing of circTP63 dramatically decreased the expression of VAPA, while the effect could be reversed through transfecting VAPA plasmids (Figure 5A). Next, CCK-8 assay was performed, and the results revealed that knockdown of circTP63 decreased the cell vitality of DU145 and PC-3 cells, while this effect could be rescued through overexpression of VAPA (Figure 5B). Moreover, transwell assay indicated that silencing of circTP63 decreased the metastasis ability of DU145 and PC-3 cells, while this effect could be reversed by overexpression of VAPA (Figure 5C). These data showed that circTP63 promotes prostate cancer progression through VAPA.

### Silencing of circTP63 restrained PCa proliferation *in vivo*

**Figure 6:**
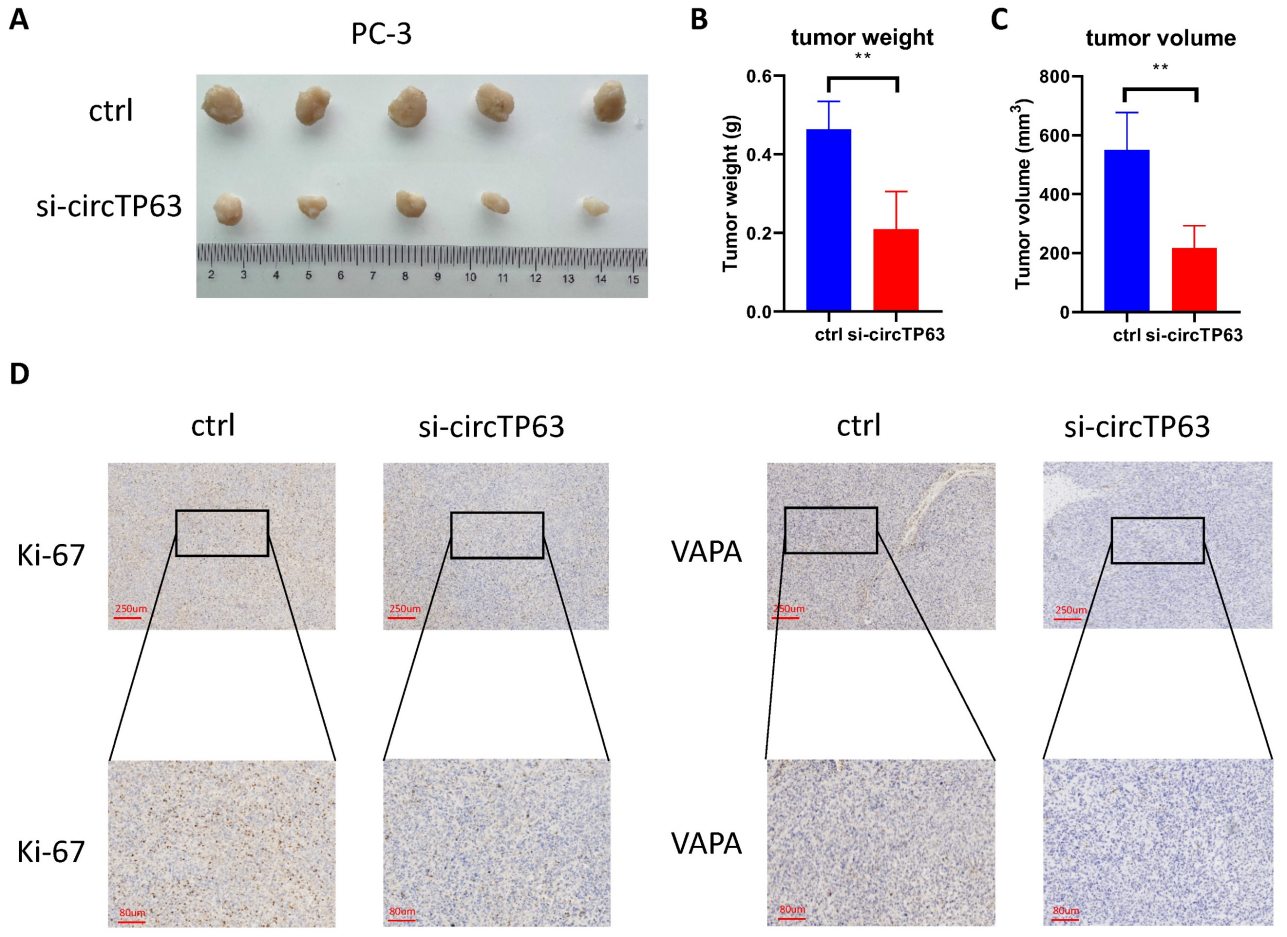
Silencing of VAPA restrains prostate cancer proliferation *in vivo*. **A-C,** The tumor weight and tumor volume were measured.** D,** Immunohistochemistry staining of Ki-67 and VAPA using subcutaneous xenograft tumor.

We further investigated the functional role of circTP63 *in vivo* using a xenograft animal model. circTP63 stably knockdown or control PC-3 cells were subcutaneously injected into nude mice. Consistent with the* in vitro* observation, silencing of circTP63 significantly reduced the tumor growth *in vivo* (Figure 6A-6C). Furthermore, IHC staining for Ki-67 and VAPA were performed in the subcutaneous tumors. Consistent with our previous results, Ki-67 and VAPA levels were reduced in tumors with circTP63 knockdown (Figure 6D). Therefore, circTP63 knockdown restrained tumor progression* in vivo*.

## Discussion

Nowadays, increasing evidences have revealed that circRNAs play a crucial role in cancer progression, including prostate cancer 25-27. Has_circRNA_102002 have been reported to promote papillary thyroid cancer metastasis via sponging miR-488-3p, and then activated HAS2 28. circHIPK3, a well-known circRNA which has been found to be differentially expressed in various tumors, promoted colorectal cancer progression through binding to miR-7 29. However, the detailed mechanism of circRNA in prostate cancer remains poorly understood. In our study, we examined the expression of circTP63 in 40 paired prostate cancer and adjacent normal tissues, and found circTP63 was upregulated in prostate cancer tissues. Then, RNase R digestion and actinomycin D treatment assays confirmed the circular characteristic of circTP63. We then explored the role of circTP63 in prostate cancer. CCK-8 assay showed that circTP63 silencing restrained the proliferation of PCa cells. Furthermore, circTP63 knockdown increased the apoptosis rate of PCa cells. Transwell assays revealed that silencing of circTP63 inhibited PCa cells metastasis. Moreover, xenograft animal model received the same results, which verified that circTP63 was an oncogenic circRNA in prostate cancer.

We then explored the detailed mechanism of circTP63 in PCa progression. Since the cellular distribution analysis showed that circTP63 was mainly distributed in the cytoplasm, we hypothesized that circTP63 may exert its function through serving as a miRNA sponge. AGO2-RIP assay showed that circTP63 could specifically bind to AGO2, suggesting that circTP63 could sponge miRNAs. Bioinformatics analysis was then performed to find the potential target miRNAs of circTP63. Dual luciferase reporter assay confirmed the interaction of circTP63 and miR-421. Moreover, correlation analysis confirmed that circTP63 negatively correlated with miR-421 in prostate cancer tissues. These data suggested that circTP63 promoted PCa progression.

miRNAs are a class of noncoding RNAs that are about 17-26 nucleotides long. miR-421 is a well-studied miRNA, which plays different roles in different types of tumors. It has been reported that miR-421 promoted gastric cancer, lung cancer, ovarian cancer, and papillary thyroid cancer progression 30-33. However, miR-421 played the opposite role in glioma, colorectal cancer, and prostate cancer 34-36. Bhatia et al. revealed that miR-421 restrained SPINK1 induced prostate cancer progression via binding to the 3'UTR of SPINK1 mRNA. In the current study, CCK-8 assay revealed that inhibition of miR-421 promoted PCa proliferation, which was in agreement with the previous results. Meanwhile, silencing of circTP63 restrained the promotion effect of miR-421 inhibition, suggesting that circTP63 promoted PCa progression via modulating miR-421 expression. By using bioinformatics analysis, VAPA was selected as the potential target of miR-421. Silencing of miR-421 elevated the expression of VAPA, while miR-421 overexpression declined VAPA expression. Also, tissue verification showed that VAPA was downregulated in prostate cancer tissues. Correlation analysis indicated a negative correlation between miR-421 and VAPA. Functional assays indicated that overexpression of VAPA could rescue the inhibitory effect of circTP63 in PCa proliferation and metastasis. In conclusion, these data revealed that circTP63 promoted PCa progression via miR-421/VAPA pathway.

Our study has several limitations: i) larger sample size is needed to explore the clinical characteristics of circTP63 in prostate cancer; ii) lack of clinical information to verify the association of circTP63 and disease prognosis; iii) although VAPA was proven to promote prostate cancer progression in our study, more attention should be focused on exploring the specific mechanism by which VAPA promotes prostate cancer progression.

## Conclusion

**Figure 7:**
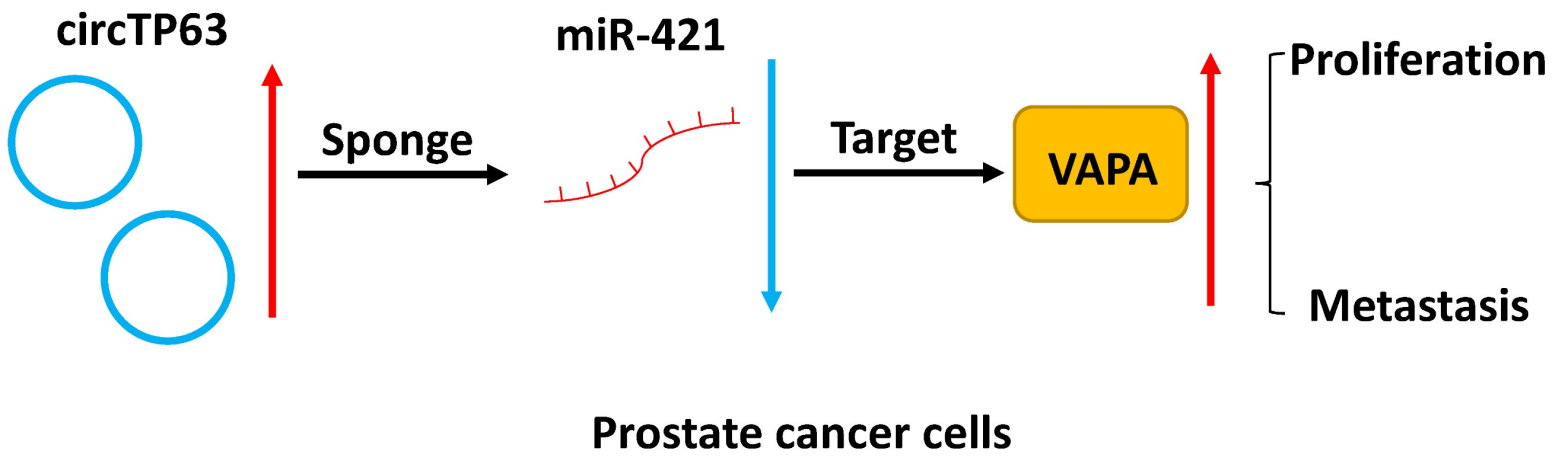
Schematic illustration of circTP63 promotes prostate cancer progression via miR-421/VAMP associated protein A axis.

In summary, our study demonstrated that circTP63 promotes prostate cancer progression via directly binding to miR-421, thus increasing the expression of VAPA. This indicates that circTP63 could be a promising diagnostic and therapeutic target for prostate cancer (Figure 7).

## Supplementary Material

Supplementary figures and table.

## Figures and Tables

**Table 1:** Relationship between circTP63 and clinicopathological characteristics in PCa patients

	circTP63 expression		
Characteristics	Low expression (N=20)	High expression (N=20)	*p* value
Age			0.4292
>55	15	17	
≤55	5	3	
T Stage			0.0181*
T1-T2	3	10	
T2-T4	17	10	
Metastasis			0.3758
Yes	2	4	
No	18	16	
Gleason score			0.0765
6-8	1	5	
9-10	19	15	

## Abbreviations

CircRNA: circular RNAs
PCa: prostate cancer
AR: androgen receptor
VAPA: VAMP associated protein A
ER: endoplasmic reticulum
ADT: androgen deprivation therapy
RIP: RNA immunoprecipitation
