# Supplementary material for: circTP63 promotes prostate cancer progression via miR-421/VAMP associated protein A axis
Source: J Cancer. 2024 Aug 19;15(16):5451–61. doi: 10.7150/jca.99561 (PMC11375539; doi:10.7150/jca.99561)
Supplement: Supplementary file 1 — Supplementary figures and table. [file jcav15p5451s1.pdf]

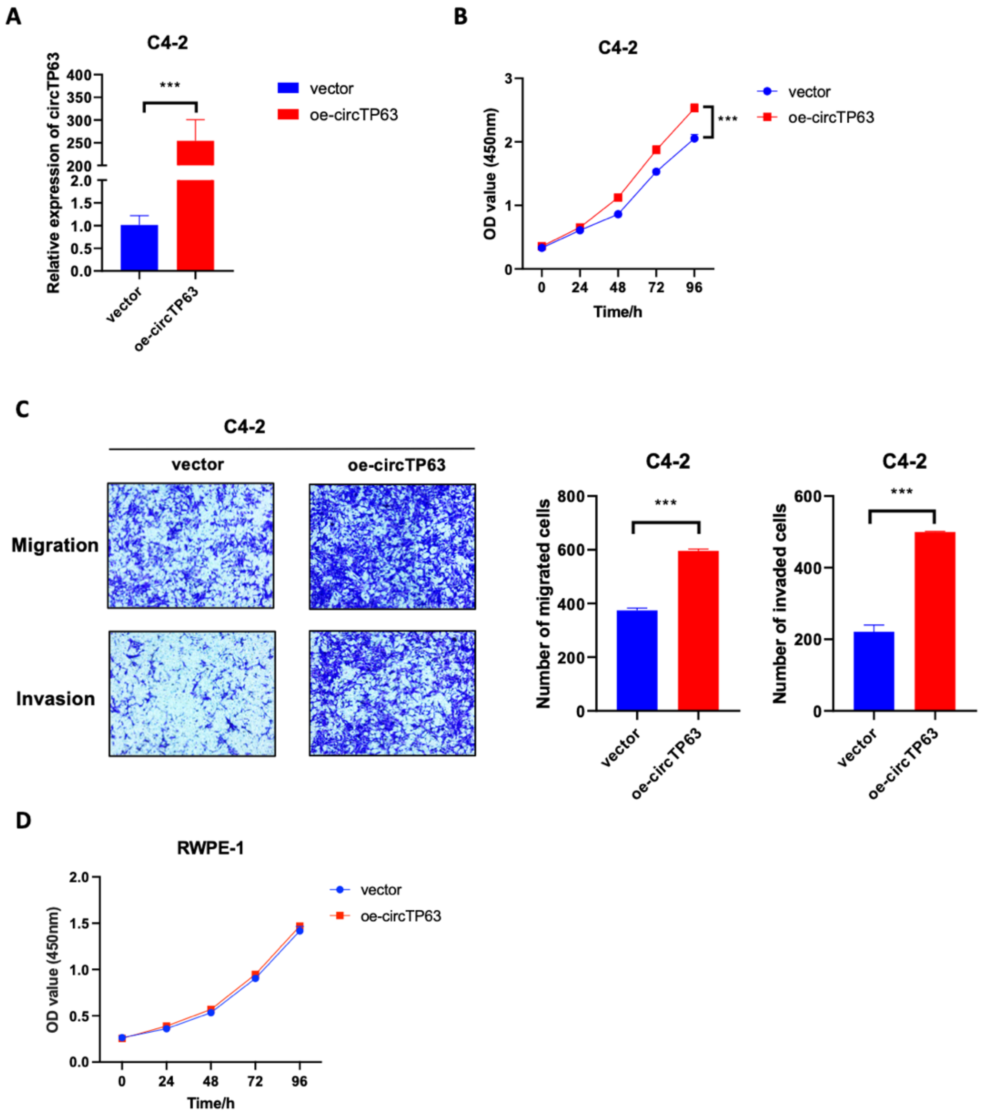

Supplementary Figure 1: **A,** The relative expression of circTP63 in C4-2 cells transfected with circTP63 overexpression plasmids. **B**, The growth curve of C4-2 cells evaluated by CCK-8 assay with circTP63 overexpression. **C,** Transwell assay in C4-2 cells with circTP63 overexpression. **D,** The growth curve of RWPE-1 cells evaluated by CCK-8 assay with circTP63 overexpression

A

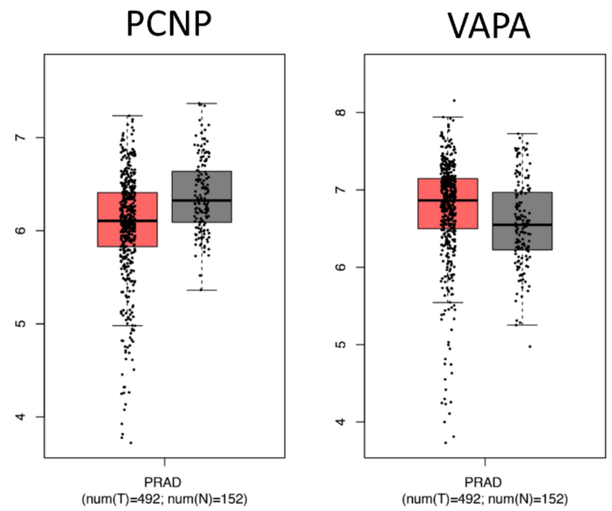

Supplementary Figure 2: **A,** The relative expression of PCNP and VAPA in TCGA database (PRAD)

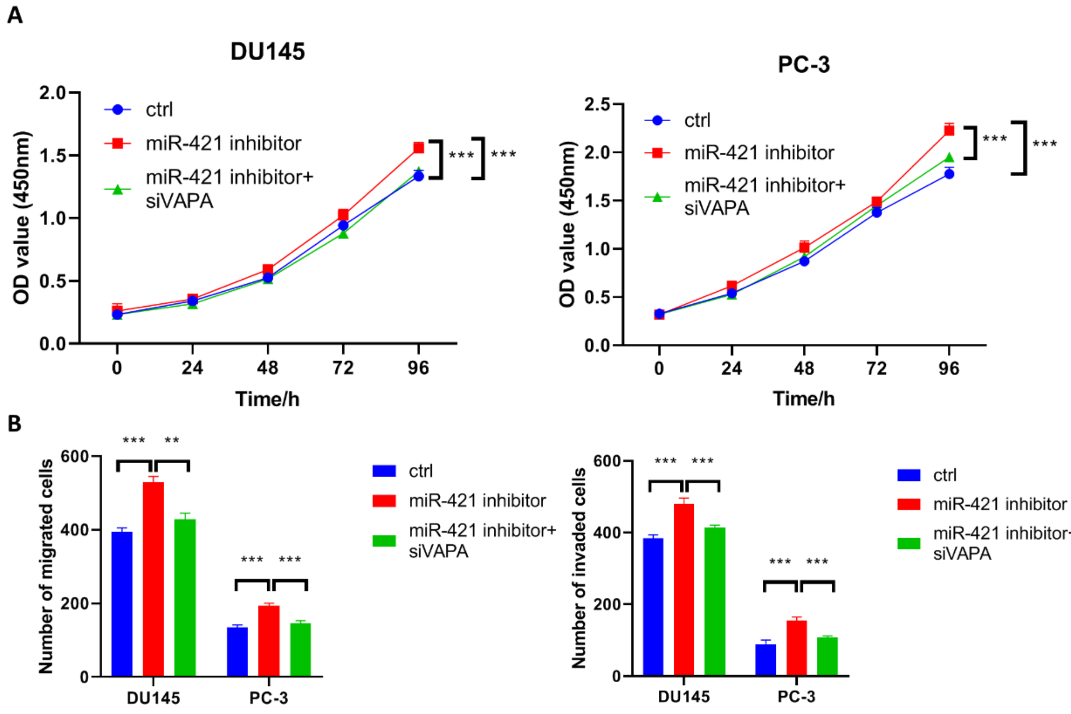

Supplementary Figure 3: **A,** The growth curve of PCa cells with VAPA knockdown reversed the promotion effect of miR-421 inhibition. **B,** Transwell assay of DU145 and PC-3 with VAPA knockdown reversed the promotion effect of miR-421 inhibition.

Table S1. Primer sequences for RT-qPCR used in this study.

Gene	Forward primer, 5' to 3'	Reverse primer, 5' to 3'
circTP63	GCCCTCACTCCTACAACCATT	TTGTGTGCTGAGGAAGGTACT
GAPDH	GGCCTCCAAGGAGTAAGACC	AGGGGAGATTCAGTGTGGTG
U6	CTCGCTTCGGCAGCACA	AACGCTTCACGAATTTGCGT

Gene	RT primer	Forward primer, 5' to 3'	Reverse primer, 5' to 3'
miR-421	GTCTCCTCTGGTGGAGGGTCCGAGGTATTCCCACCAGAGGAGACGCGCCC	GCGCGGATCAACAGACATTAATT	GTGCAGGGTCCGAGGT
